# Long noncoding RNA *Gm31629* promotes bone regeneration by maintaining bone marrow mesenchymal stem cells activity

**DOI:** 10.7717/peerj.13475

**Published:** 2022-06-09

**Authors:** Guangping Cai, Ye Xiao, Mi Yang, Qi Guo, Tian Su, Yalin Liu, Tiejian Jiang, Chun Li

**Affiliations:** 1 Department of Endocrinology, Endocrinology Research Center, Xiangya Hospital of Central South University, Changsha, Hunan, China; 2 National Clinical Research Center for Geriatric Disorders, Xiangya Hospital, Changsha, Hunan, China

**Keywords:** Long noncoding RNA Gm31629, BMSCs, Senescence, Bone regeneration, YB-1

## Abstract

**Background:**

Long noncoding RNA *Gm31629* can regulate hypothalamic neural stem cells (htNSCs) senescence and the aging process. However, the effect of *Gm31629* on the senescence of bone marrow mesenchymal stem cells (BMSCs) and bone regeneration is unclear. In the present study, we investigated the effects of *Gm31629* on the senescence of BMSCs and bone regeneration.

**Methods:**

*Gm31629* knockout (*Gm31629*-KO) and wild-type (WT) mice were used to establish a bone regeneration model. The Brdu labelling, CCK8 assay, wound healing assay, β-gal staining and osteogenic differentiation assay were used to assess the effects of *Gm31629* on the functions of BMSCs. Micro-computed tomography (CT), histochemical and immunohistochemical staining were used to evaluate the ability of bone regeneration. The mimic of *Gm31629*, theaflavin 3-gallate, was used to investigate its role on the senescence of BMSCs and bone regeneration.

**Results:**

The expression of *Gm31629* reduced in BMSCs of middle-aged mice was compared with that of young mice. The deletion of *Gm31629* was sufficient to drive the senescence of BMSCs, resulting in impaired bone regeneration in mice. Mechanistically, *Gm31629* could interact with Y-box protein 1(YB-1) and delay its degradation, decreasing the transcription of *p16^INK4A^* of BMSCs. We also found that theaflavin 3-gallate could alleviate the senescence of BMSCs and promote bone regeneration in middle-aged mice.

**Conclusion:**

These results indicated that *Gm31629* played an important role on BMSCs senescence and bone regeneration and provided a therapeutic target to promote bone regeneration.

## Introduction

Bones, the primary structural material of mammals, are often damaged throughout life and undergo constant modeling, remodeling and repair (Borrelli Jr et al., 2012; Taylor, Hazenberg & Lee, 2007; Zheng et al., 2019). Bone is a powerful self-healing tissue, but the ability to self-heal in the elderly can be reduced by complex changes at the molecular, cellular, and systemic levels (Gruber et al., 2006). Bone repair is a complex biological process involving the synergistic participation of vascular and skeletal precursor cells within the bone marrow (Dimitriou, Tsiridis & Giannoudis, 2005).

Bone marrow mesenchymal stem cells (BMSCs), which can self-renew and differentiate into multiple cell types, make great contributions to the regeneration of mesenchymal tissues such as cartilage, adipose and bone (Lu, Li & Cheng, 2002; Pittenger et al., 1999). Moreover, it has been suggested that BMSCs can act as potent microenvironmental regulators, which exert influence on various tissues, including bone (Liu et al., 2015; Su et al., 2019; Sudres et al., 2006; Xiao et al., 2021; Xu et al., 2018b; Yu et al., 2021). For example, Yu et al. (2021) reported that BMSCs-derived exosomal miR-136-5p promoted osteoblast proliferation, differentiation, thus facilitating fracture healing. Accordingly, BMSCs have been widely used in bone regeneration including bone tissue engineering for their close involvement in bone formation (Fernandes & Yang, 2016; Lu, Li & Cheng, 2002; Zhang et al., 2019). With age, various cell types, including BMSCs, undergo senescence (Aguayo-Mazzucato et al., 2019; Wang et al., 2020; Wiley et al., 2021). Senescent BMSCs not only showed decreased ability to differentiate to osteoblasts, but also showed a declining capacity for proliferation and migration (Geissler et al., 2012; Li et al., 2015; Moerman et al., 2004; Sethe, Scutt & Stolzing, 2006). Moreover, exosomal miR-31a-5p secreted by senescent BMSCs can not only inhibit osteogenic differentiation, but also promote osteoclast differentiation (Xu et al., 2018b). All of these may lead to impaired therapeutic effects of senescent BMSCs in bone regeneration. However, the exact mechanism of BMSCs senescence remains unclear.

Long non-coding RNAs (LncRNAs), which are characterized by transcripts more than 200 nucleotides in length, play a variety of regulatory roles through interactions with DNA, RNA and proteins (Huo et al., 2018). They have been observed to participate in the regulation of many biological processes and diseases, involving cell senescence, apoptosis, differentiation, proliferation, and tumorigenesis (Guo et al., 2020; Klattenhoff et al., 2013; Ng, Johnson & Stanton, 2012; Xiao et al., 2020; Yang et al., 2011). Recently, several studies have revealed that lncRNAs are involved in regulating osteogenic differentiation of BMSCs and bone repair (Liu et al., 2022; Ouyang et al., 2020). Our previous study showed that lncRNA, *Gm31629* is down-regulated in the hypothalamic neural stem cell (htNSCs) of middle-aged mice compared with that of young mice (Xiao et al., 2020). Deletion of *Gm31629* accelerated the senescence of htNSCs and leaded to aging-associated phenotype in mice (Xiao et al., 2020). *Gm31629* could regulate the senescence of htNSCs by delaying the degradation of YB-1 (Xiao et al., 2020). YB-1 is a DNA/RNA-binding protein (Lyabin, Eliseeva & Ovchinnikov, 2014) and has been reported to bind to the promoter region of *p16*^*INK*4*A*^ and inhibit its expression (Kotake et al., 2013; Xiao et al., 2020), a maker of cellular senescence (Ogrodnik et al., 2019; Omori et al., 2020). However, the role of *Gm31629* in the senescence of BMSCs and bone regeneration has not been investigated.

In the present study, we expanded our research and demonstrated that *Gm31629* could also regulate the senescence of BMSCs and bone regeneration. Deletion of *Gm31629* accelerated the degradation of YB-1, promoted the senescence of BMSCs, and impaired the ability of bone regeneration. We also found that the natural compound, theaflavin 3-gallate (TF2A), could mimic the activity of *Gm31629* and alleviate the senescence of BMSCs. Treatment of TF2A could promote bone regeneration in middle-aged mice.

## Materials and Methods

### Animals and treaments

*Gm31629* knockout (*Gm31629-KO*) mice were obtained from Cyagen Biosciences as previously reported (Xiao et al., 2020). We purchased 3-month-old and 12-month-old C57BL/6J male mice from the Laboratory Animal Center of Central South University (Changsha, China). The model of bone regeneration was established as described before (Chen et al., 2019; Fukuda et al., 2013; Yang et al., 2020). Briefly, after anesthesia, the anterior medial approach was used to expose the distal femoral. Then, a 25-guage needle was used to drill a hole at the distal femur along the long axis of the femur and a 0.6 mm diameter Kirschner wire was used to ablate trabecular bone of distal femur. This was minimally invasive injury and we made great effort to reduce the sufferings of the mice. One week after the injury, the mice were euthanized by cervical dislocation after anesthesia to collect the bone samples.

For TF2A administration, mice were treated with TF2A or vehicle by gavage at a dosage of 8 mg/kg every day for three weeks before the establishment of bone regeneration model. After that, TF2A treatment continued for one week before the mice were euthanized. All the mice in this study were healthy and C57BL/6 background and kept in the Experimental Animal Research Center of Central South University with specific pathogen-free standard. The mice were housed in individual ventilated cage with six mice per cage. The mice were kept in room temperature with 12 h light-dark cycle and had free access to food and water. No animal was excluded from the experiments. Xiangya Hospital of Central South University Ethics Committee (Changsha, Hunan, China) approved this research (2019030350). All animal experiments conformed to all ethical requirements relating to animal research.

### BMSCs isolation, culture and senescence assays

BMSCs were isolated as previously described (Li et al., 2015; Yu et al., 2018). The isolated BMSCs were cultured with α-MEM supplemented with 15% FBS, 100 U/mL penicillin and 100 µg/mL streptomycin in a humidified atmosphere of 5% CO2 at 37 °C to reach 80% confluence. Then the first-passage BMSCs were harvested and seeded in culture dishes for enrichment of cell populations. When the second-passage reach confluence after 1–2 week, they were subcultured. Only third-passage BMSCs were applied to perform further study unless specified otherwise.

The senescent BMSCs were stained by a senescence β-galactosidase staining Kit (Solarbio Science & Technology) according to the manufacturer’s instructions. Briefly, after washing with PBS, the cells were fixed with 4% paraformaldehyde for 15 min at room temperature. Then the cells were stained with working solution overnight at 37 °C. Five different fields were randomly selected under a microscope to count the SA-βGal-positive (blue cells) and the percentage of SA-βGal-positive were calculated.

### Cell transfection

For *Gm31629* overexpression, the adenovirus particles expressing mouse *Gm31629* were purchased from OBiO Technology Corporation (Shanghai, China). For Yb-1 overexpression, pcDNA3.1-mYb-1 was purchased from Sino Biological Inc (Beijing, China). PcDNA3.1-mYb-1 and negative control were transfected into BMCSs with lipofectamine 2000 (Invitrogen, Thermo Fisher Scientific, Waltham, MA) by a standard method.

### Wound healing assay

BMSCs were seeded in 6-well plates at a density of 1 ×10^6^ cells per well for each group. A linear wound was made using a sterile 200 µl pipette tip to scratch across the confluent cell layer. Images of wound healing were observed at 0 h and 24 h and the migration rate was calculated using ImageJ software (National Institutes of Health, Bethesda, MD, USA).

### CCK8 assay

CCK8 assay was used to evaluate the growth of BMSCs according to the manufacture’s protocol (MedChemExpress, LLC). BMSCs were seeded in 96-well plates at a density of 5,000 cells per well for each group. Then, we added 10 µl of CCK-8 solution into each well and incubated the plate at 37 °C for 2 h. At last, the OD value of each well was examined at 450 nm using a spectrophotometer (Thermo Fisher Scientific, Waltham, MA, USA).

### Brdu staining assay

Brdu staining assay was conducted using standard methods. Briefly, BMSCs were seeded in 24-well plates at a density of 1 × 10^5^ cells per well for each group and incubated with 10 µM Brdu labeling solution for 24 h in a cell incubator. After that, the cells were fixed with 4% paraformaldehyde for 20 min. After permeabilization using 0.2% triton, the cells were incubated with 3% BSA for blocking. Then, the cells were incubated with the primary (Cell Signaling Technology, Danvers, MA; 5292, 1:400) and secondary antibody (Invitrogen, Thermo Fisher Scientific, Waltham, MA; A21202, 1:500), and counterstained DAPI.

### Osteogenic differentiation assay

BMSCs were seeded in 6-well plates at a density of 5 × 10^5^ cells per well for each group and cultured with osteogenic induction conditional medium (10 mM β-glycerol phosphate, 50 µM ascorbate-2-phosphate, and 0.1 µM dexamethasone) for three weeks. We changed the osteogenic medium every other day. To assess the mineralization of cell matrix, 2% Alizarin Red S (Cyagen Biosciences, Santa Clara, CA) was used to stain the cell matrix. Alizarin Red S was destained with cetyl-pyridinium chloride solution and the OD value was quantified by spectrophotometry at 562 nm.

### Osteoclast differentiation assay

We perform osteoclasts differentiation assay as described before (Yang et al., 2020). Briefly, bone marrow was flushed out of bone marrow cavity of mice. Isolated bone marrow cells were cultured with complete media for 14 h. Then the unattached cells were collected and treated with α-MEM containing 10% FBS, 30 ng/mL M-CSF (R&D Systems Inc., Minneapolis, MN), 100 µg/mL streptomycin, and 100 U/mL penicillin for 72 h to gain pure monocytes and macrophages. After that, the monocytes and macrophages were cultured with osteoclastic induction medium (30 ng/mL M-CSF, 60 ng/mL RANKL) for 1 week. Osteoclasts were stained with TRAP staining kit (Sigma-Aldrich, St Louis, MO) according to manufacturer’s instructions.

### RT-qPCR analysis

Extraction of total RNA was performed with Trizol (Invitrogen, Thermo Fisher Scientific, Waltham, MA) following standard methods and reverse transcription was conducted using 1 µg total RNA. RT-qPCR was conducted in duplicate using SYBR Premix Ex Taq II (Takara). We normalized the Ct value of *Gm31629* to that of *Gapdh* and calculated ΔCt value (ΔCt=Ct_(*Gm*31629)_- Ct_(*Gapdh*)_) for both 3-moth-old and 12-moth-old group. Then we normalized the ΔCt value of 12-moth-old group to the ΔCt value of 3-moth-old group and calculated ΔΔCt value (ΔΔCt = Δ *Ct*_(12-moth-old)_−Δ *Ct*_(3-moth-old)_). The relative gene expression was calculated using the 2^−ΔΔCT^ method. All experiments were repeated three times. The primer sequences are listed in Table S1.

### Western blot

Western blotting was performed as previously described (Liu et al., 2021b; Peng et al., 2019). Total cell proteins were separated by SDS-PAGE and blotted on PVDF membranes (Millipore, Sigma, Burlington, MA). After blocking with 5% milk, the membranes were incubated with specific antibodies to YB-1 (Cell Signaling Technology, Danvers, MA; 4202, 1:1000), p16^INK4A^ (Sigma-Aldrich, St Louis, MO, SAB4500072, 1:1000) and GAPDH (Proteintech, Rosemont, IL, USA; 10494-1-AP, 1:5000). Blots were visualized using an ECL Kit (Thermo Fisher Scientific, Waltham, MA; 32,106).

### RNA pull-down assay and RNA immunoprecipitation

We performed RNA pull down assay as previously reported (Xiao et al., 2020). Briefly, biotin-labeled full-length *Gm31629* and antisense *Gm31629* were incubated with nuclear lysate of BMSCs for 1 h. After that, the streptavidin agarose beads (Invitrogen, Thermo Fisher Scientific, Waltham, MA) were added and incubated at 25 °C for another 1 h. After washing with cold NT2 buffer, the pulled-down proteins were used for western blot analysis. RNA immunoprecipitation was performed using a Magna RIP RNA-Binding Protein Immunoprecipitation Kit (Millipore, Sigma, Burlington, MA; 17-700) following the manufacturer’s instructions. The precipitated RNA was extracted, reversed transcribed and analyzed by RT-qPCR. All experiments were repeated three times. The primer sequences are listed in Table S1.

### Chromatin immunoprecipitation (ChIP) assay

ChIP assay was performed with SimpleChip Kit (9003; Cell Signaling Technology, Danvers, MA) following the manufacturer’s instructions as previously described (Yang et al., 2019). Briefly, chromatin was crossed-linked (1 % formaldehyde, 10 min) and sheared to 100- to 500-bp fragments by sonication. The relevant protein-DNA complex was immunoprecipitated by YB-1 antibody (Santa Cruz Biotechnology, Dallas, TX; sc-398146) or IgG control. The ChIP DNA was used to perform standard PCR or RT-qPCR. All experiments were repeated three times. The primer sequences are listed in Table S1.

### µCT analysis

µCT scanning was performed using a high-resolution micro-CT (SCANCO Medical AG, VIVACT 80; Wangen-Brüttisellen, Switzerland) with a resolution of 12 µm per pixel at 55 kV and 145 µA. We reconstructed a 3D model and analyzed the structure indices as previous reported (Li et al., 2021; Yang et al., 2019; Yang et al., 2020). Trabecular bone volume (Tb. BV/TV) in the regeneration region was calculated.

### Histochemistry and immunohistochemistry

Histochemical and immunohistochemical staining were conducted as previously described (Cai et al., 2022; Yang et al., 2020). Femora were collected and fixed with 4% paraformaldehyde for 1 day at 4 °C. Then we decalcified the bones with 10% EDTA and embedded them in paraffin. For histochemistry, 4 micrometer-thick slides were subjected to HE and TRAP staining according to a standard protocol. For immunocytochemistry, after antigen retrieval the samples were incubated with primary antibodies against osteocalcin (Takara M173) at 4 °C overnight and Horseradish peroxidase-streptavidin detection system (Dako Agilent, Santa Clara, CA) was used to detect immuno-activity.

### Statistics analysis

Statistical analysis was performed using GraphPad Prism software 8.0. Data are expressed as the mean ± standard deviation (sd). Unpaired Student’s *t* test was applied to compare two groups. One-way ANOVA was employed while comparing multiple groups. The difference was considered to be statistically significant at *p* < 0.05. In order to avoid the type II error, we used G*Power 3.1 to perform the statistical power analysis and the minimum power required in this study was set at 0.8. All the samples were randomly assigned and no blinding was used.

## Results

### BMSCs undergo senescence during aging with reduced ability of bone regeneration

**Figure 1:**
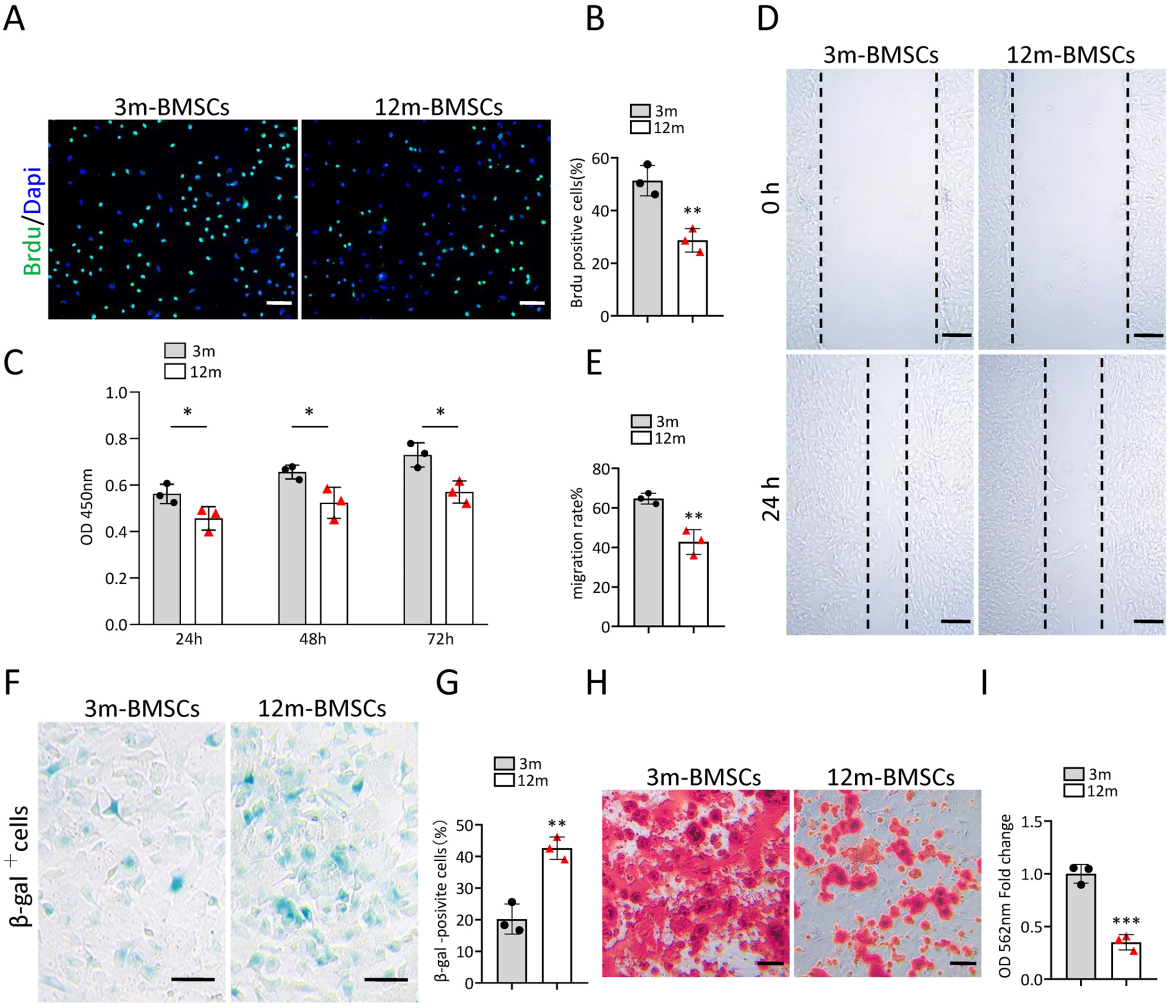
The increased cell senescence in BMSCs of middle-aged mice. (A) Representative images of Brdu assay. Scale bar:100 µm. (B) Quantification of Brdu positive cells. (*n* = 3). (C) CCK8 assay (*n* = 3). (D) Representative images of BMSCs migration in wound healing test. Scale bar:200 µm. (E) Quantitative analysis of migration rate. (*n* = 3). (F) SA-βGal staining of BMSCs. Scale bar: 50 µm. (G) The percentage of SA-βGal positive cells. (*n* = 3). (H) ARS staining of BMSCs under osteogenic induction. Scale bar: 100 µm. (I) Quantification of calcium mineralization (*n* = 3). Data are expressed as mean ± sd and statistical differences were analyzed by Student’s *t* test. ^∗^*p* < 0.05; ^∗∗^*p* < 0.01; ^∗∗∗^*p* < 0.001.

To study the characteristics of senescent BMSCs, we compared the function and phenotype of BMSCs from middle-aged (12-month old) mice with that from young mice (3-month old) *in vitro*. The Brdu staining assay and CCK8 assay revealed that the proliferation ability of BMSCs from middle-aged mice was significantly reduced in comparison with BMSCs from young mice (Figs. 1A–1C). The wound healing assay indicated that the migration ability of BMSCs from middle-aged mice was markedly declined in comparison with BMSCs from young mice (Figs. 1D, 1E). As expected, there were more SA-βGal-positive BMSCs in the middle-aged group than in the young groups (Figs. 1F, 1G). BMSCs from middle-aged mice also showed decreased osteogenic differentiation ability compared to that of young mice (Figs. 1H, 1I). These results indicated that an aging phenotype of BMSCs presented in the middle-aged mice, resulting in significant impaired function of BMSCs.

**Figure 2:**
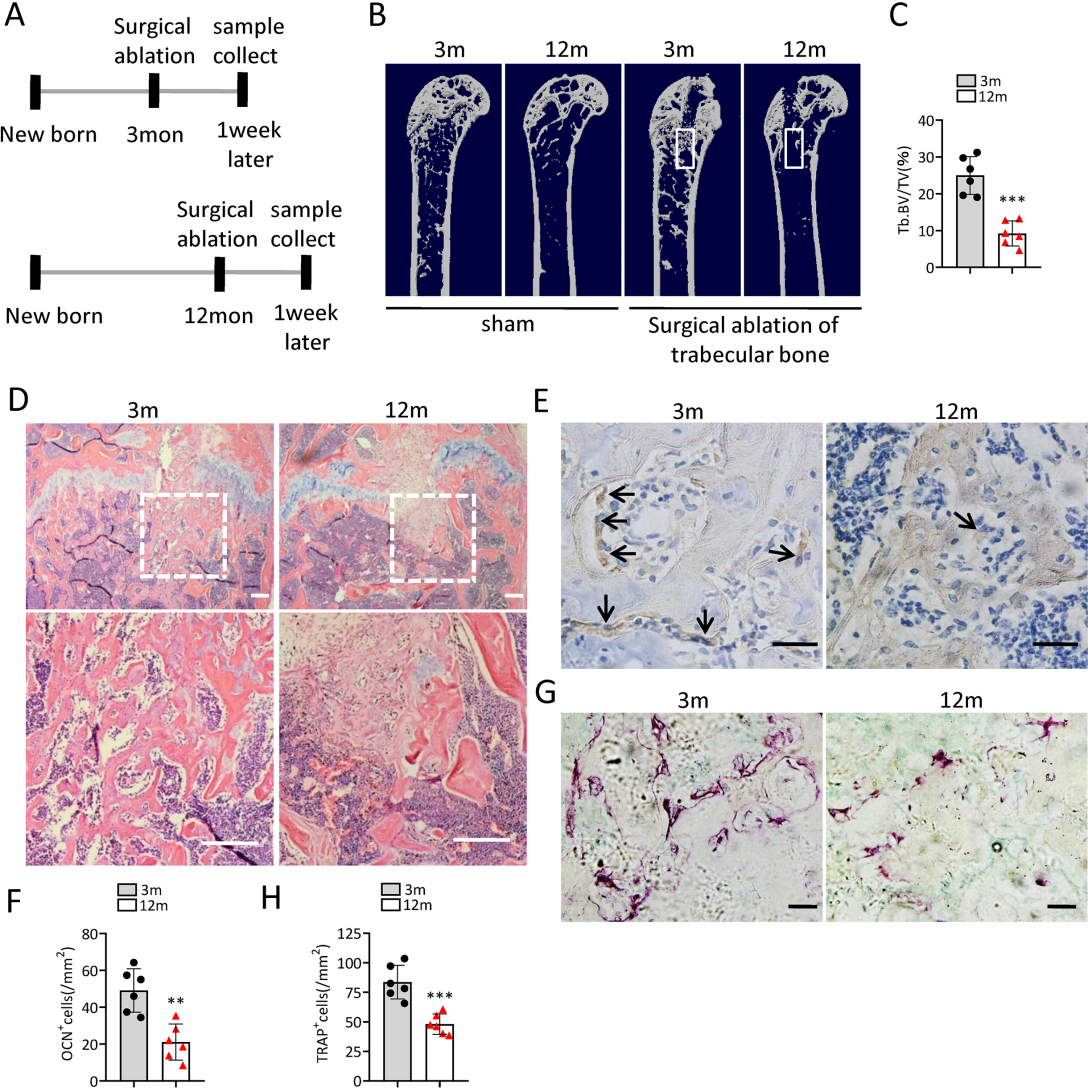
The ability of bone regeneration decreases during aging. (A) Time plan for surgical ablation of trabecular bone in distal femoral of mice. (B) Representative micro-CT images. The white square was selected to measure trabecular bone volume in bone regeneration region. (C) Quantification of trabecular bone volume in bone regeneration region. (*n* = 6). (D) HE staining of distal femora. Scale bar: 200 µm. (E) Immunohistochemical staining of osteocalcin positive cells. Black arrows represent osteocalcin positive cells. Scale bar: 50 µm. (F) Quantitative analysis of osteocalcin positive cells. (*n* = 6). (G) TRAP staining images. Scale bar: 50 µm. (H) Quantitative analysis of TRAP positive cells. (*n* = 6). Data are expressed as mean ± sd and statistical differences were analyzed by Student’s *t* test. ^∗∗^*p* < 0.01; ^∗∗∗^*p* < 0.001.

To investigate the change of bone regeneration ability during aging, a bone regeneration model was established by surgical ablation of trabecular bone in distal femur. As expected, the bone volume in the regeneration area of middle-aged mice was lower than that of young mice at 7 days after ablation (Figs. 2A–2D). The number of osteocalcin positive (ocn^+^) osteoblasts in the bone regeneration area of middle-aged mice was less than that of young mice at 7 days after ablation (Figs. 2E, 2F). The number of tartrate-resistant acid phosphatase positive (TRAP^+^) osteoclasts in the bone regeneration area of middle-aged mice was also less than that of young mice at 7 days after ablation (Figs. 2G, 2H). Altogether, these results indicated that middle-aged mice had reduced bone regeneration ability compared to young mice.

### *Gm31629* regulates the senescence of BMSCs

**Figure 3:**
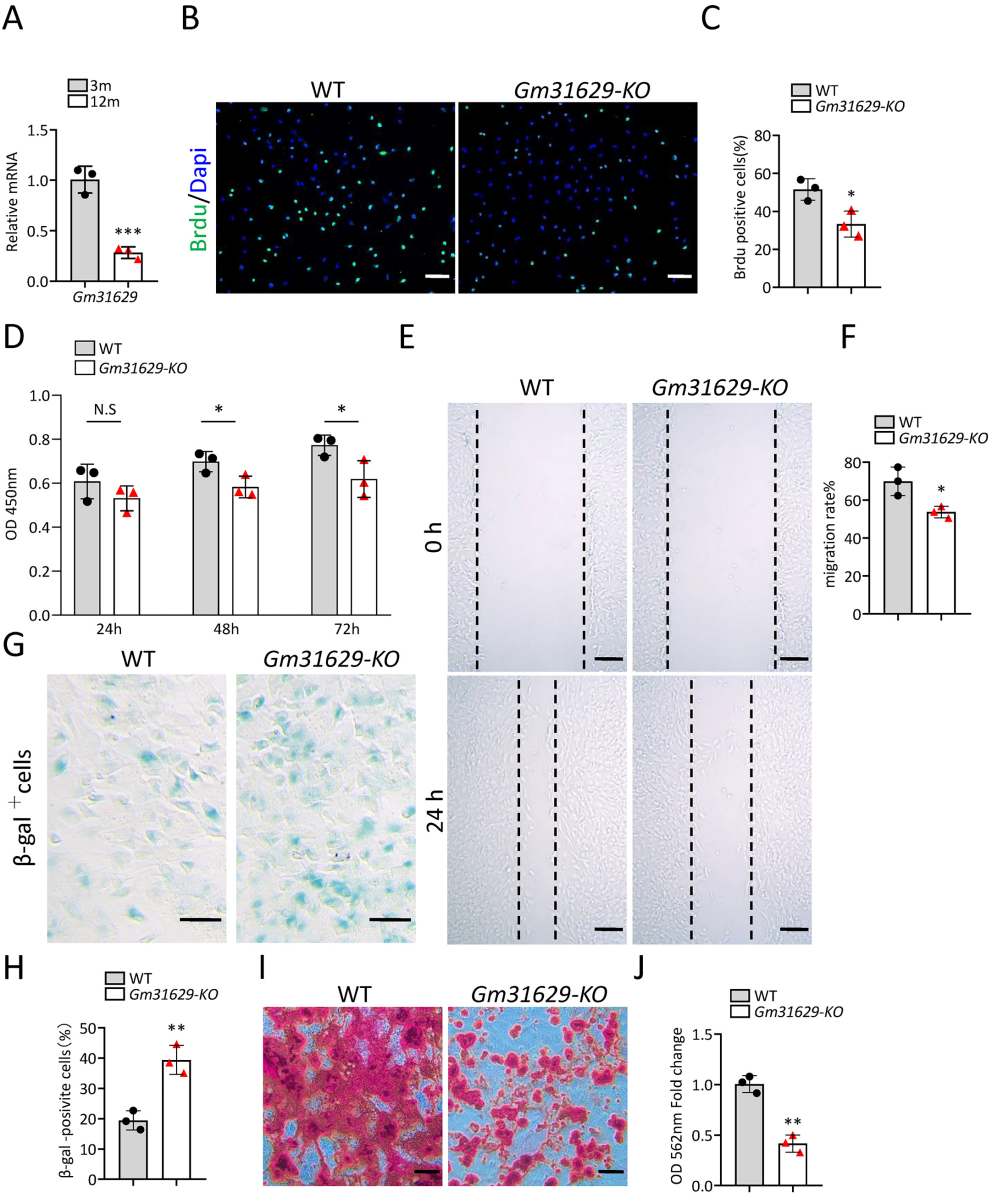
*Gm31629* regulates the senescence of BMSCs. (A) Relative *Gm31629* expression in BMSCs of 3-month-old and 12-month-old mice as analyzed by RT-qPCR. The expression level of *Gm32129* in BMSCs of 3-month-old mice was set at an arbitrary value = 1. (*n* = 3). (B) Representative images of Brdu assay. Scale bar: 100 µm. (C) Quantification of Brdu positive cells. (*n* = 3). (D) CCK8 assay. (*n* = 3). (E) Representative images of BMSCs migration in wound healing test. Scale bar: 200µm. (F) Quantitative analysis of migration rate. (*n* = 3). (G) SA-βGal staining of BMSCs. Scale bar: 50 µm. (H) The percentage of SA-βGal positive cells. (*n* = 3). (I) ARS staining of BMSCs under osteogenic induction. Scale bar: 100 µm. (J) Quantification of calcium mineralization. (*n* = 3). Data are expressed as mean ±sd and statistical differences were analyzed by Student’s *t* test. ^∗^*P* < 0.05; ^∗∗^*P* < 0.01; ^∗∗∗^*P* < 0.001; N.S, no significance.

*Gm31629* expression decreased significantly in BMSCs of middle-aged mice compared with that of young mice as analyzed by RT-qPCR (Fig. 3A). To study the function of *Gm31629* in the regulation of BMSCs senescence, we isolated BMSCs from 3-month-old *Gm31629* knockout (*Gm31629-KO*) mice and wild type (WT) mice. The proliferation and migration ability of BMSCs isolated from *Gm31629-KO* mice were markedly reduced in comparison with that of BMSCs isolated from WT mice (Figs. 3B–3F). In addition, there were more SA-βGal-positive BMSCs in *Gm31629-KO* group than in WT group (Figs. 3G, 3H). BMSCs of *Gm31629-KO* mice also showed decreased osteogenic differentiation capacity compared to that of WT group (Figs. 3I, 3J). These data revealed that BMSCs from *Gm31629-KO* mice showed an aging phenotype with significant impaired function of BMSCs.

### *Gm31629* knockout mice show impaired bone regeneration ability

**Figure 4:**
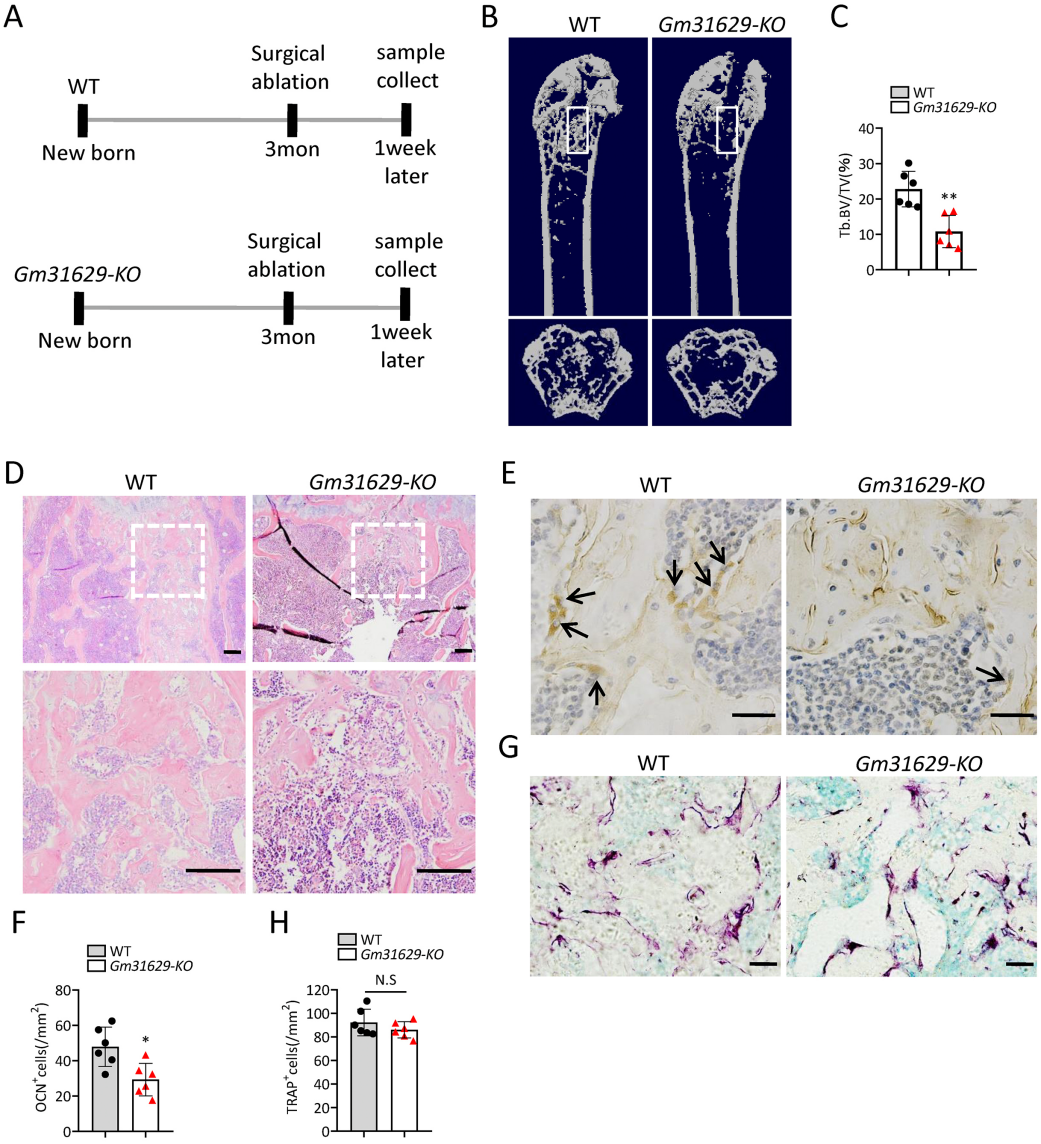
*Gm31629-KO* mice show impaired ability of bone regeneration. (A) Time plan for surgical ablation of trabecular bone in distal femoral of mice. (B) Representative micro-CT images. The white square was selected to measure trabecular bone volume in bone regeneration region. (C) Quantification of trabecular bone volume in bone regeneration region. (*n* = 6). (D) HE staining of distal femora. Scale bar: 200 µm. (E) Immunohistochemical staining of osteocalcin positive cells. Black arrows represent osteocalcin positive cells. Scale bar: 50 µm. (F) Quantitative analysis of osteocalcin positive cells. (*n* = 6). (G) TRAP staining images. Scale bar: 50 µm. (H) Quantitative analysis of TRAP positive cells. (*n* = 6). Data are expressed as mean ± sd and statistical differences were analyzed by Student’s *t* test. ^∗^*P* < 0.05; ^∗∗^*P* < 0.01; N.S, no significance.

To further study the function of *Gm31629* in bone regeneration, we established the bone regeneration model in *Gm31629-KO* mice and WT mice at 3-month-old by surgical ablation of trabecular bone in distal femur (Fig. 4A). We found that the bone volume in bone regeneration region of *Gm31629-KO* mice was significantly lower than that of WT controls at 7 days after ablation (Figs. 4B–4D). The number of ocn^+^ osteoblasts was markedly reduced in bone regeneration region of *Gm31629-KO* mice in comparison with that of WT controls at 7 days after ablation (Figs. 4E, 4F). There is no significant difference of TRAP^+^ osteoclasts in bone regeneration region of *Gm31629-KO* mice in comparison with that of WT controls 7 days after ablation (Figs. 4G, 4H). These data showed that the bone regeneration ability of *Gm31629-KO* mice was lower than that of WT mice.

### *Gm31629* regulates BMSCs senescence through YB-1/P16^*INK*4*A*^ pathway

**Figure 5:**
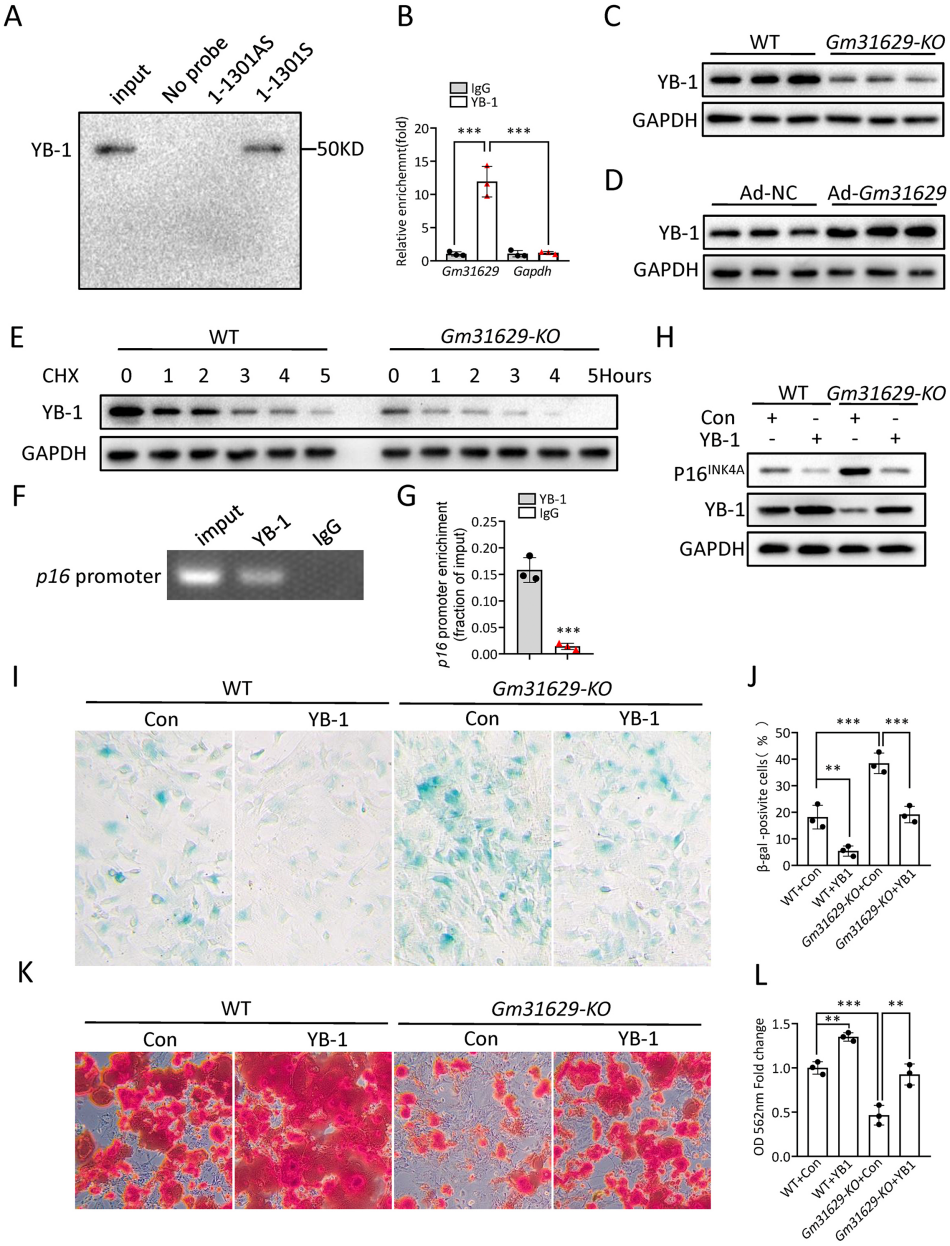
*Gm31629* regulates BMSCs senescence through YB-1/P16^INK4A^ pathway. (A) Western blot analysis of YB-1 pulled-down by *Gm31629* (1-1301S) and antisense *Gm31629* (1-1301AS) or other controls. (B) YB-1-retrieved *Gm31629* RNA as determined by RT-qPCR analysis. The level of IgG-retrieved *Gm31629* was set at an arbitrary value = 1. (C) Western blotting analysis of YB-1 protein in WT and *Gm31629-KO* BMSCs. (D) Western blotting analysis of YB-1 protein in adenovirus vector-driven *Gm31629* overexpressed or control BMSCs. (E) Western blotting analysis of YB-1 protein in WT and *Gm31629-KO* BMSCs treated with CHX. (F) The binding of YB1 to the *p16^INK4A^* promoter was detected by ChIP -PCR assay with an antibody against YB1 or IgG. (G) The abundance of YB-1 binding on the promoter of *p16^INK4A^* was determined by ChIP assay followed by RT-qPCR analysis. (H) Western blotting result of YB-1 and P16^INK4A^ protein in WT and *Gm31629-KO* BMSCs with or without YB-1 overexpressed. (I) Representative images of SA-βGal staining of BMSCs. Scale bar:50 µm.(J) The percentage of SA-βGal positive cells. (*n* = 3). (K) ARS staining of BMSCs under osteogenic induction. Scale bar:100 µm. (L) Quantification of calcium mineralization. (*n* = 3). Data are expressed as mean ± sd and statistical differences were analyzed by Student’s *t* test or one-way ANOVA. ^∗∗^*P* < 0.01; ^∗∗∗^*P* < 0.001.

Our previous study demonstrated that *Gm31629* directly interacted with YB-1 and increased the protein level of YB-1 by preventing its degradation, further reducing the expression of *p16*^*INK*4*A*^ and suppressing the senescence of htNSCs (Xiao et al., 2020). YB-1 is a DNA/RNA-binding protein (Lyabin, Eliseeva & Ovchinnikov, 2014), and has been reported to bind to the promoter region of *p16*^*INK*4*A*^ and inhibit the expression of *p16*^*INK*4*A*^ (Kotake et al., 2013; Xiao et al., 2020), a maker of cellular senescence (Ogrodnik et al., 2019; Omori et al., 2020). Furthermore, several studies have demonstrated that the expression of *p16*^*INK*4*A*^ is much higher in BMCSs of older mice than in young controls (Hu et al., 2022; Li et al., 2017). To verify the interaction between YB-1 and *Gm31629* in BMSCs, the RNA pull-down assay was repeated and the binding of *Gm31629* to YB-1 in BMSCs was confirmed (Fig. 5A). RNA immunoprecipitation assay further confirmed that *Gm31629* could bind to YB-1 in BMSCs (Fig. 5B). In addition, *Gm31629* knockout markedly reduced YB-1 protein level and *Gm31629* overexpression significantly increased YB-1 protein level (Figs. 5C, 5D). To confirm that *Gm31629* increases YB-1 protein level by preventing the degradation of YB-1 in BMSCs, we inhibited protein synthesis in BMSCs with cycloheximide (CHX) and found that *Gm31629* knockout accelerated the degradation of YB-1 (Fig. 5E). These results indicated that *Gm31629* could prevent the degradation of YB-1 in BMSCs. Moreover, ChIP-PCR assays showed that YB-1 could directly bind to the promoter of *p16*^*INK*4*A*^ in BMSCs (Fig. 5F, 5G). Then, we observed that *Gm31629* knockout not only reduced YB-1 protein level, but also increased the expression of *p16*^*INK*4*A*^ (Fig. 5H). However, the overexpression of YB-1 in *Gm31629* knockout BMSCs rescued the reduced YB1 protein level and reduced the expression of *p16*^*INK*4*A*^ (Fig. 5H). Moreover, the overexpression of YB-1 rescued the increased level of senescence in BMSCs derived from *Gm31629- KO* mice (Figs. 5I, 5J). In addition, osteogenic differentiation assay revealed that YB-1 overexpression rescued the reduced osteogenic differentiation of BMSCs derived from *Gm31629- KO* mice (Figs. 5K, 5L). Thus, these results indicated *Gm31629* could stabilized YB1 protein and inhibited the expression of *p16*^*INK*4*A*^, a possible mechanism for *Gm31629* in regulating the senescence of BMSCs.

### TF2A treatment *in vitro* alleviates the senescence of BMSCs

**Figure 6:**
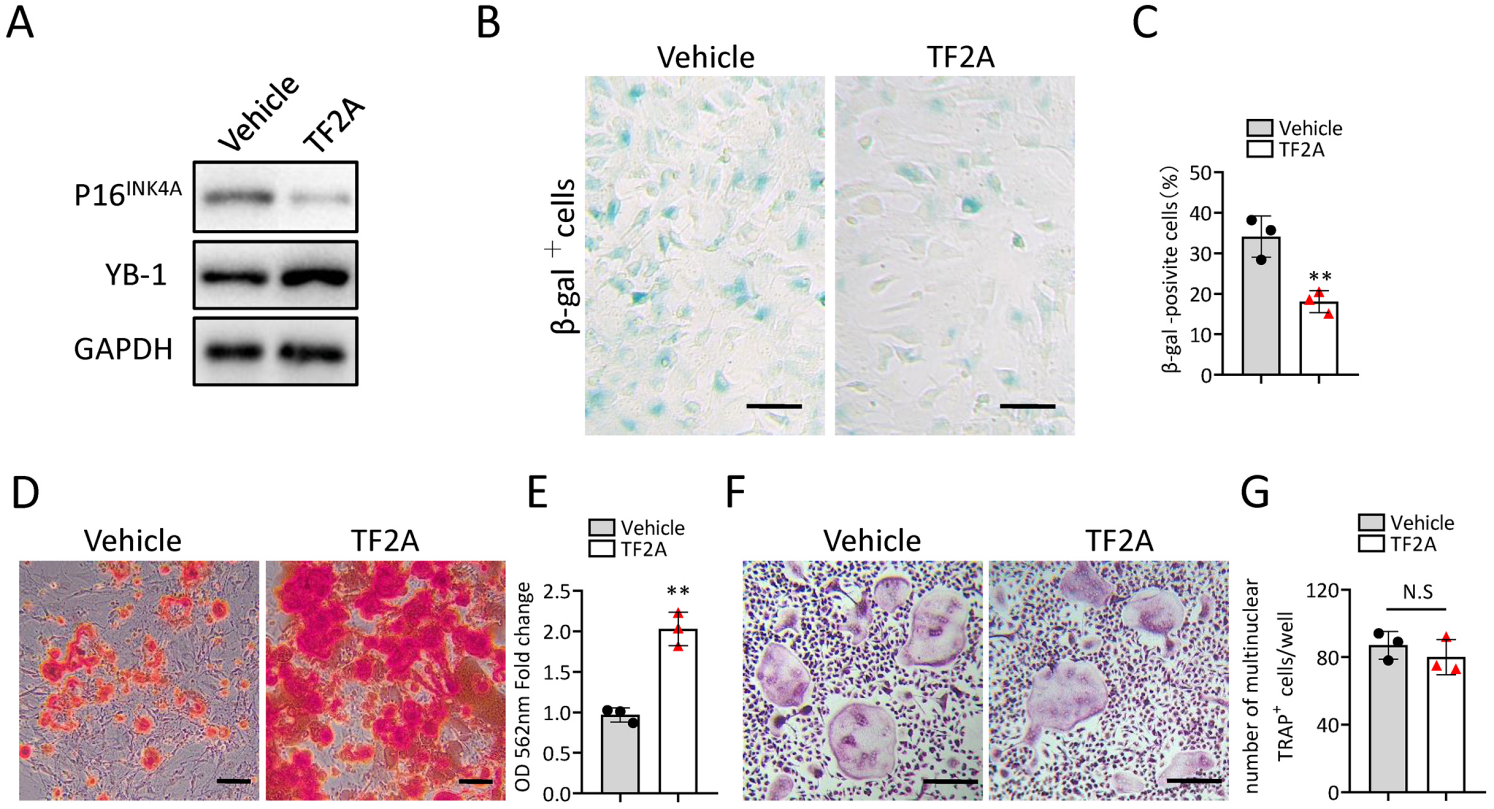
TF2A treatment *in vitro* alleviates BMSCs senescence. (A) YB-1 and P16^INK4A^ protein levels in BMSCs treated with vehicle or TF2A. (B) Representative images of SA-βGal staining of BMSCs treated with TF2A or vehicle. Scale bar: 50 µm. (C) The percentage of SA-βGal positive cells. (*n* = 3). (D) ARS staining of BMSCs under osteogenic induction. Scale bar: 100 µm. (E) Quantification of calcium mineralization. (*n* = 3). (F) TRAP staining of bone marrow monocytes and macrophages under osteoclast differentiation. Scale bar: 200 µm. (G) Quantification of multinuclear TRAP positive cells per well. (*n* = 3). Data are expressed as mean ± sd and statistical differences were analyzed by Student’s *t* test. ^∗∗^*P* < 0.01; N.S, no significance.

In the previous study, we also identified a natural compound, TF2A, which mimics the ability of *Gm31629* to increased YB-1 protein level, reducing the senescence of htNSCs (Xiao et al., 2020). As expected, treatment of TF2A could mimic the ability of *Gm31629* to increase YB-1 protein level and reduce the expression of *p16*^*INK*4*A*^ in BMSCs (Fig. 6A). We then found that TF2A attenuated the senescence of BMSCs and promoted osteogenesis of BMSCs (Figs. 6B–6E). TF2A did not affect osteoclast differentiation, as evaluated by TRAP staining (Figs. 6F, 6G). These data demonstrated that TF2A could mimic the activity of *Gm31629* to increase the protein level of YB-1, thus alleviating the senescence of BMSCs.

### TF2A treatment promotes bone regeneration in middle-aged mice

**Figure 7:**
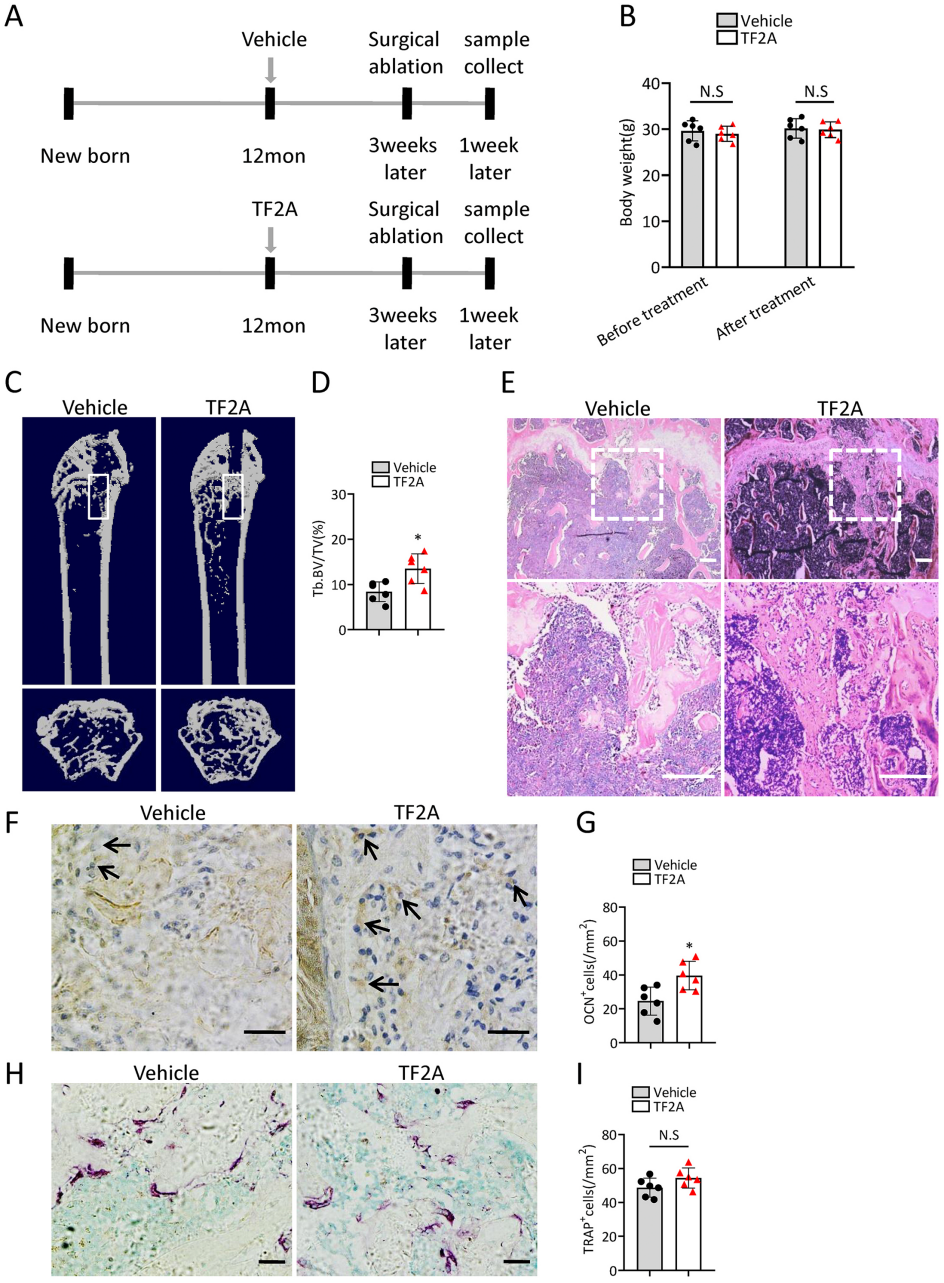
TF2A treatment promotes bone regeneration in middle-aged mice. (A) Time point at which mice were treated with TF2A or vehicle, and performed with surgical ablation of trabecular bone in distal femoral . (B) The body weight of 12-month-old mice before and after treated with TF2A or vehicle. (*n* = 6). (C) Representative micro-CT images. The white square was selected to measure trabecular bone volume in bone regeneration region. (D) Quantification of trabecular bone volume in bone regeneration region. (*n* = 6). (E) HE staining of distal femora of middle-aged mice. Scale bar: 200 µm. (F) Immunohistochemical staining of osteocalcin positive cells. Black arrows represent osteocalcin positive cells. Scale bar: 50 µm. (G) Quantitative analysis of osteocalcin positive cells. (*n* = 6). (H) TRAP staining images. Scale bar: 50 µm. (I) Quantitative analysis of TRAP positive cells. (*n* = 6). Data are expressed as mean ± sd and statistical differences were analyzed by Student’s *t* test. ^∗^*P* < 0.05; N.S, no significance.

To investigate whether treatment of TF2A could promote bone regeneration in middle-aged mice, 12-month-old C57BL/6J mice were orally treated with TF2A at a dosage of 8 mg/kg every day or with vehicle for three weeks. There weeks after TF2A or vehicle treatment, the mice were performed with surgical ablation of trabecular bone in the right femur, and continued the treatment with TF2A for one week (Fig. 7A). Administration of TF2A had no obvious influence on the weight of the mice (Fig. 7B). Treatment with TF2A promoted bone regeneration in 12-month-old mice in comparison with the control group (Figs. 7C–7E). The number of ocn+ osteoblasts in bone regeneration region was also increased after TF2A administration, which indicated increased bone formation (Figs. 7F, 7G). There was no significant difference of TRAP+ osteoclasts in bone regeneration region of TF2A treated group compared with that of vehicle treated group (Figs. 7H, 7I). These data indicated that TF2A could promote bone regeneration in middle-aged mice.

## Discussion

Bone has natural healing ability that is sufficient to repair bone injuries and the capacity of bone repair is compromised during aging (Lin et al., 2019). BMSCs are capable of self-renewal and can differentiate into various tissues, and the therapeutic potential of BMSCs for bone repair has been widely accepted (Park et al., 2012; Squillaro, Peluso & Galderisi, 2016). However, BMSCs undergo senescence during aging and show an obvious impairment in their proliferation, migration and differentiation ability (Li et al., 2017; Sepulveda et al., 2014; Xu et al., 2018b). In addition, senescent cells can secret substantial chemokines, proinflammatory cytokines, proteases, and other factors (Xu et al., 2018a; Xu et al., 2015). These factors are termed the senescence associated secretory phenotype (SASP) (Xu et al., 2015), which may contribute to impaired therapeutic effects of senescent BMSCs (Sepulveda et al., 2014; Turinetto, Vitale & Giachino, 2016). In this study, we showed that there was increased senescent BMSCs from 12-month-old mice in comparison with that from 3-month-old mice. BMSCs from 12-month-old mice exhibited an obvious impairment in their proliferation, migration and osteoblastic differentiation ability. Compared with 3-month-old mice, 12-month-old mice had compromised bone regeneration ability, accompanied by reduced osteoblast in bone regeneration area. Accordingly, the prevention of BMSCs senescence or rejuvenation of aged BMSCs is a promising strategy to improve bone regeneration.

Recently, multiple studies have focused on the mechanism of BMSCs senescence (Guo et al., 2021; Hu et al., 2022; Liu et al., 2021a). The emerging roles of lncRNAs in regulating cellular senescence have also been documented in previous studies (Lee et al., 2020; Xia et al., 2017). Our previous studies demonstrated that *Gm31629* could regulate the senescence of htNSCs, and loss of *Gm31629* accelerated aging-like phenotype (Xiao et al., 2020). Here, we extended our research and demonstrated that *Gm31629* could also regulate the senescence of BMSCs and bone regeneration. BMSCs from *Gm31629-KO* mice showed a premature aging phenotype and their proliferation, migration, and osteogenic differentiation abilities were reduced. *Gm31629-KO* mice had compromised bone regeneration ability with reduced osteoblast in bone regeneration area. We did not observe significant changes in osteoclast between *Gm31629-KO* mice and WT mice, which indicated *Gm31629* had no effect on osteoclasts. Previously, Sun et al. (2019) reported that lncRNA *lnc-ob1* could regulate osteoblast activity and bone formation *via* upregulating the expression of *Osterix* in osteoblast. Since *Gm31629 -KO* mice are global *Gm31629* knockout and the compromised bone regeneration ability of *Gm31629 -KO* mice may also result from loss of function of osteoblasts, osteocytes or other bone cells. A tissue-specific mouse model will be more convincing to elucidate the role of *Gm31629* in regulating BMSCs senescence and bone regeneration.

At the mechanistic level, we found that *Gm31629* regulated the senescence of BMSCs and bone regeneration *via* interacting with YB-1 protein to delay its degradation. YB-1 is a multifunctional protein that can bind RNA and DNA (Lyabin, Eliseeva & Ovchinnikov, 2014). By binding to nucleic acids, YB-1 participates in basic gene expression process, including transcription, mRNA stabilization and translation (Lyabin, Eliseeva & Ovchinnikov, 2014). At the cellular level, YB-1 has been reported to regulate a variety of biological activities including cell proliferation, differentiation, senescence and apoptosis (Kotake et al., 2013; Lyabin, Eliseeva & Ovchinnikov, 2014). For example, Kotake et al. (2013) demonstrated that YB-1 could bind to the promoter region of *p16*^*INK*4*A*^, inhibit its expression and prevent cellular senescence. In this study, our results confirmed that YB-1 could bind to *p16*^*INK*4*A*^ promoter, repress the expression of *p16*^*INK*4*A*^ and prevent BMSC senescence. These findings suggest that *Gm31629*-YB-1 signaling axis plays a critical role in BMSC senescence and bone regeneration.

Previously, Evans et al. (2020) reported that YB-1 could fine-tunes Polycomb repressive complex2 (PRC2) activities to control embryonic neural development. The findings of Schmid et al. (2013) suggested YB-1 could act as a mediator of Melanoma inhibitory activity (MIA)/cartilage-derived retinoic acid-sensitive protein (CD/RAP) dependent chondrogenesis. These studies suggest that *Gm31629*-YB-1 signaling axis may also affect chondrogenesis and neurogenesis of BMSCs, which requires further study.

TF2A is one of the isomeric monomers of black tea theaflavins and theaflavins have been reported to have many beneficial effects for the health (Anandhan et al., 2012; Lin, Huang & Lin, 2007; Tong et al., 2018; Zhang et al., 2016). Previously, we identified that TF2A could mimic the activity of *Gm31629* and reduce the senescence of htNSCs, thus further alleviating age-related physiological decline (Xiao et al., 2020). In this study, we further demonstrated that TF2A also could alleviate the senescence of BMSCs and improve bone regeneration ability of middle-aged mice. Consistent with the function of *Gm31629*, TF2A had no obvious effects on osteoclasts.

**Figure 8:**
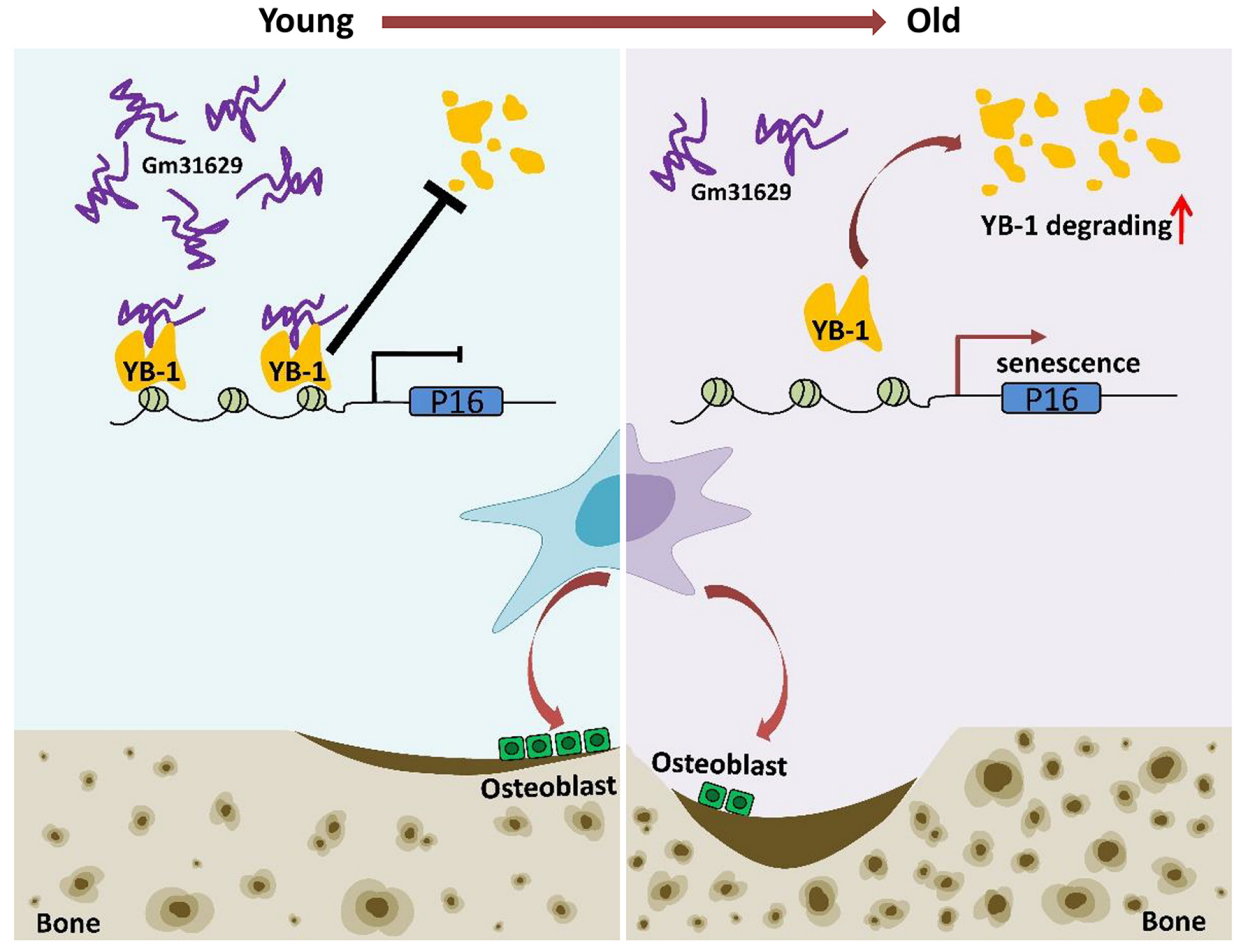
Schematic representation of *Gm31629* regulating BMSCs senescence and bone regeneration. *Gm31629* interacts with YB-1 and delays its degradation, thus decreasing the transcription of *p16^INK4A^* and suppressing the senescence of BMSCs. In old subjects, the decreased expression of *Gm31629* drives the senescence of BMSCs and leads to impaired bone regeneration.

In summary, we showed the important role of *Gm31629* in regulating BMSCs senescence and bone regeneration. *Gm31629* could interact with YB-1 and delay its degradation, thus decreasing the transcription of *p16*^*INK*4*A*^ and suppressing the senescence of BMSCs (Fig. 8). Hence, this study provides a potential new approach to attenuate BMSCs senescence and improve bone regeneration ability in aged subjects.

## Supplemental Information

10.7717/peerj.13475/supp-1 Supplemental Information 1 Primer sequence used for qRT-PCR and ChIP assays

10.7717/peerj.13475/supp-2 Supplemental Information 2 Raw data

10.7717/peerj.13475/supp-3 Supplemental Information 3 Uncropped WB image

10.7717/peerj.13475/supp-4 Supplemental Information 4 Checklist

## Additional Information and Declarations

### Data Availability

The following information was supplied regarding data availability: The raw measurements are available in the Supplementary File.
