# Supplementary material for: Long noncoding RNA Gm31629 promotes bone regeneration by maintaining bone marrow mesenchymal stem cells activity
Source: PeerJ. 2022 Jun 9;10:e13475. doi: 10.7717/peerj.13475 (PMC9188769; doi:10.7717/peerj.13475)
Supplement: Supplemental Information 1 [file peerj-10-13475-s001.docx]

Supplementary table 1. Primer sequence used for qRT-PCR and ChIP assays.

| gene | Primer sequence (5’-3’) |
| --- | --- |
| **For RT-qPCR** |  |
| *Gm31629*(mouse) | F: CTAAGCGAACTCGGGAGC |
|  | R: CACAGCAGGATTGATGGATG |
| *Gapdh* (mouse) | F: TGTGTCCGTCGTGGATCTGA |
|  | R: CCTGCTTCACCACCTTCTTGA |
| **For ChIP-PCR** |  |
| *p16*(mouse) | F: ACGTGTGCACTTCTTTGCTG |
|  | R: CATAGGTGGCGCTATTTGC |

F, forward primer; R, reverse primer; RT-qPCR, real-time quantitative PCR; ChIP assays, chromatin immunoprecipitation assays
