# Supplementary figures and images for: Long noncoding RNA Gm31629 promotes bone regeneration by maintaining bone marrow mesenchymal stem cells activity
Source: PeerJ. 2022 Jun 9;10:e13475. doi: 10.7717/peerj.13475 (PMC9188769; doi:10.7717/peerj.13475)

Figure 5

A

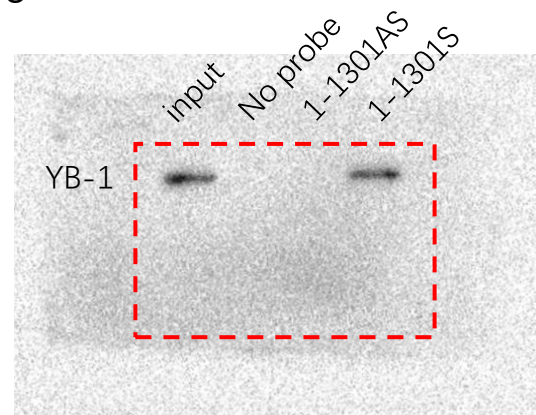

Figure 5

C

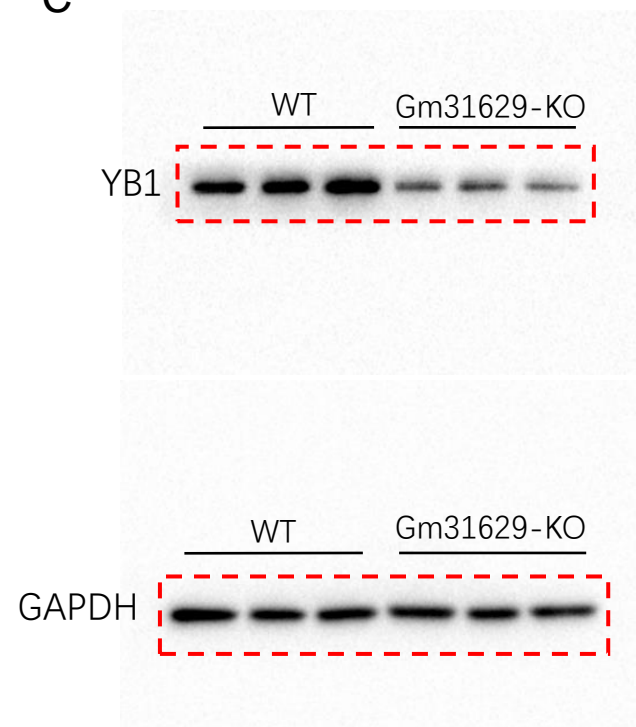

Figure 5

D

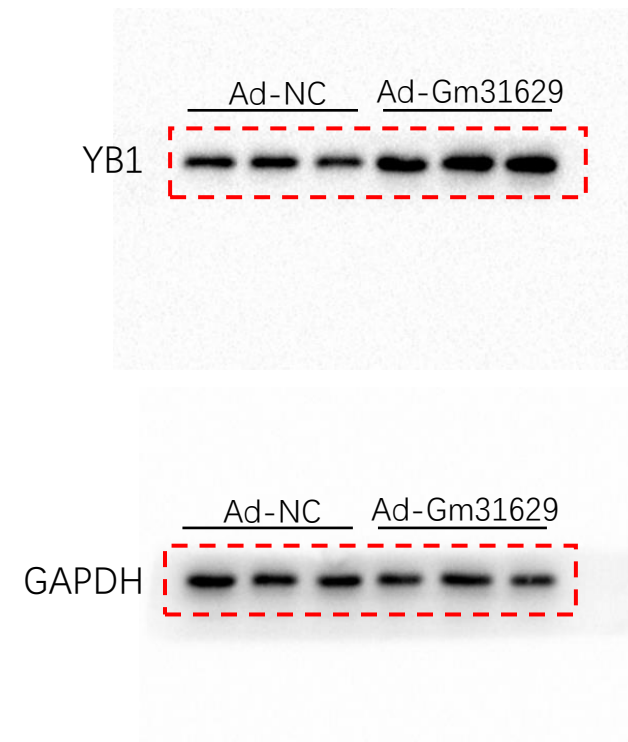

Figure 5

E

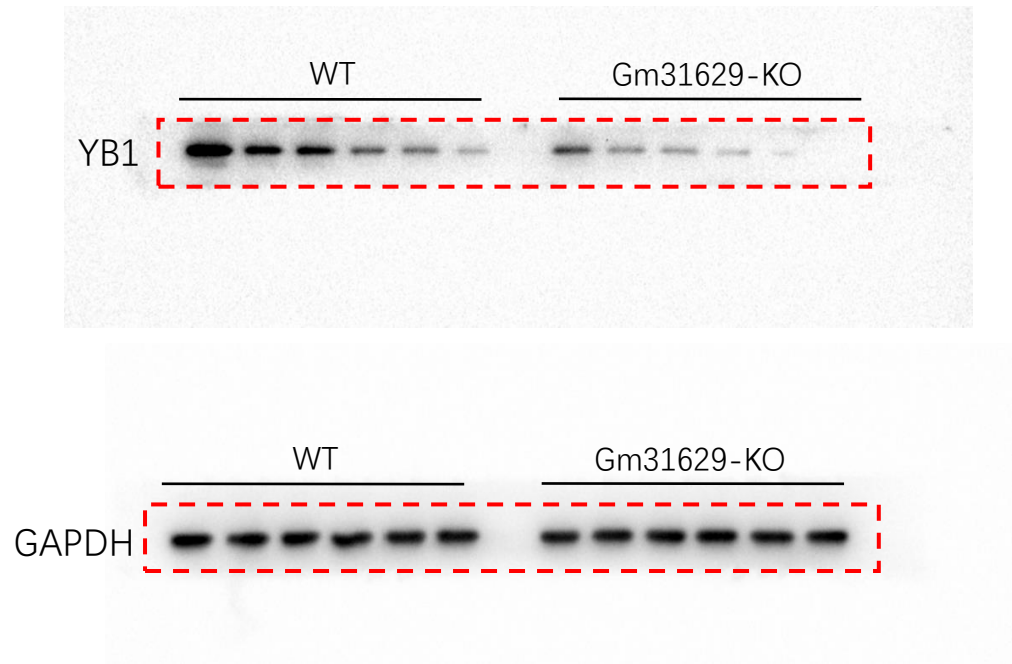

Figure 5

H

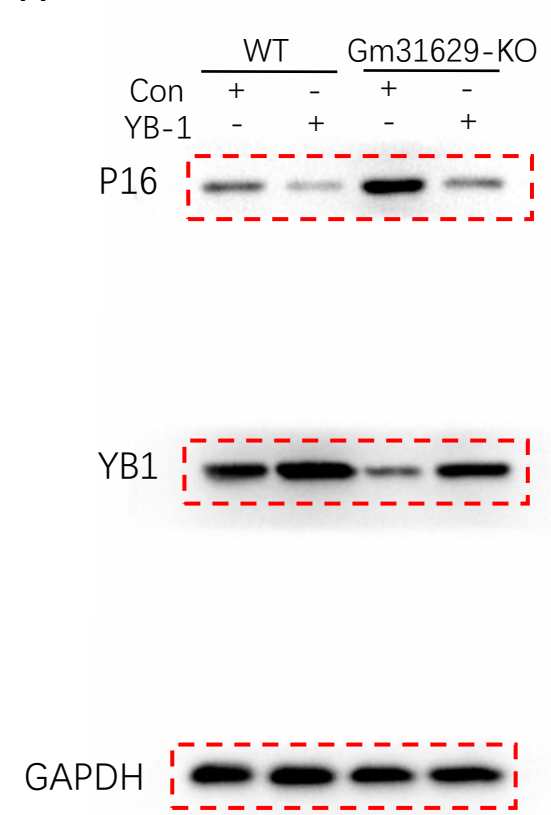

Figure 6

A

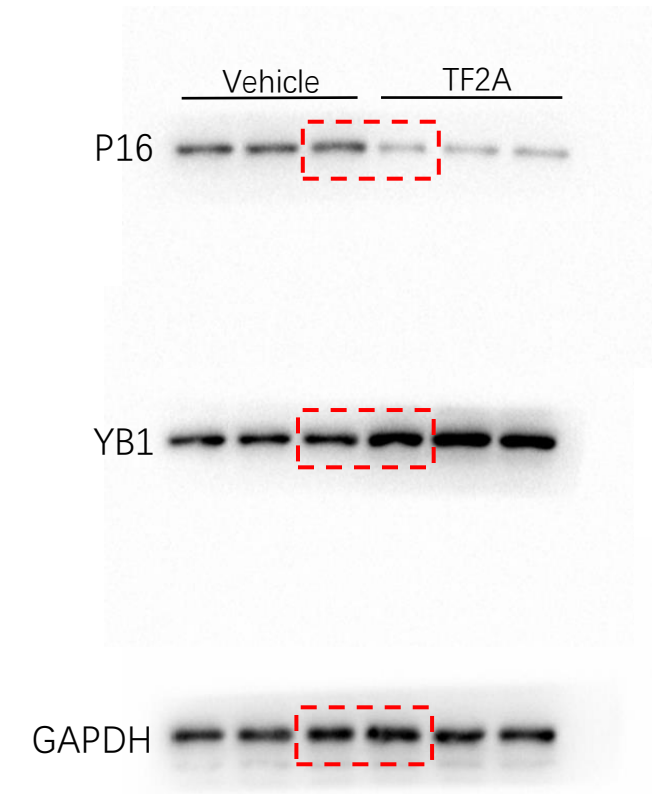

Supplement: Supplemental Information 3 [file peerj-10-13475-s003.zip › uncropped WB.pdf]
